# Cotargeting of Mitochondrial Complex I and Bcl-2 Shows Antileukemic Activity against Acute Myeloid Leukemia Cells Reliant on Oxidative Phosphorylation

**DOI:** 10.3390/cancers12092400

**Published:** 2020-08-24

**Authors:** Fangbing Liu, Hasini A. Kalpage, Deying Wang, Holly Edwards, Maik Hüttemann, Jun Ma, Yongwei Su, Jenna Carter, Xinyu Li, Lisa Polin, Juiwanna Kushner, Sijana H. Dzinic, Kathryn White, Guan Wang, Jeffrey W. Taub, Yubin Ge

**Affiliations:** 1 National Engineering Laboratory for AIDS Vaccine, Key Laboratory for Molecular Enzymology and Engineering, School of Life Sciences, Jilin University, Changchun 130012, China; liufb18@mails.jlu.edu.cn (F.L.); majun17@mails.jlu.edu.cn (J.M.); suy@karmanos.org (Y.S.); lixy17@mails.jlu.edu.cn (X.L.); 2 Center for Molecular Medicine and Genetics, Wayne State University School of Medicine, Detroit, MI 48201, USA; hkalpage@med.wayne.edu (H.A.K.); mhuttema@med.wayne.edu (M.H.); 3 The Tumor Center of the First Hospital of Jilin University, Changchun 130021, China; DeYingW@hotmail.com; 4 Department of Oncology, Wayne State University School of Medicine, Detroit, MI 48201, USA; pitmanh@karmanos.org (H.E.); polinl@karmanos.org (L.P.); kushnerj@karmanos.org (J.K.); dzinics@karmanos.org (S.H.D.); whitek@karmanos.org (K.W.); 5 Molecular Therapeutics Program, Barbara Ann Karmanos Cancer Institute, Wayne State University School of Medicine, Detroit, MI 48201, USA; 6 Cancer Biology Graduate Program, Wayne State University School of Medicine, Detroit, MI 48201, USA; jenna.carter2@med.wayne.edu; 7 Division of Pediatric Hematology/Oncology, Children’s Hospital of Michigan, Detroit, MI 48201, USA; jtaub@med.wayne.edu; 8 Department of Pediatrics, Wayne State University School of Medicine, Detroit, MI 48201, USA

**Keywords:** acute myeloid leukemia, Bcl-2, venetoclax, IACS-010759, oxidative phosphorylation

## Abstract

Targeting oxidative phosphorylation (OXPHOS) is a promising strategy to improve treatment outcomes of acute myeloid leukemia (AML) patients. IACS-010759 is a mitochondrial complex I inhibitor that has demonstrated preclinical antileukemic activity and is being tested in Phase I clinical trials. However, complex I deficiency has been reported to inhibit apoptotic cell death through prevention of cytochrome c release. Thus, combining IACS-010759 with a BH3 mimetic may overcome this mechanism of resistance leading to synergistic antileukemic activity against AML. In this study, we show that IACS-010759 and venetoclax synergistically induce apoptosis in OXPHOS-reliant AML cell lines and primary patient samples and cooperatively target leukemia progenitor cells. In a relatively OXPHOS-reliant AML cell line derived xenograft mouse model, IACS-010759 treatment significantly prolonged survival, which was further enhanced by treatment with IACS-010759 in combination with venetoclax. Consistent with our hypothesis, IACS-010759 treatment indeed retained cytochrome c in mitochondria, which was completely abolished by venetoclax, resulting in Bak/Bax- and caspase-dependent apoptosis. Our preclinical data provide a rationale for further development of the combination of IACS-010759 and venetoclax for the treatment of patients with AML.

## 1. Introduction

While overall survival rates of patients with acute myeloid leukemia (AML) have improved over the past several decades, improvement has been incremental [1]. Although the majority of AML patients treated with standard induction chemotherapy initially respond, there is a high rate of relapse due to the survival of some leukemia initiating/stem cells [2,3]. Further, older adults (>60 years old), who make up the majority of AML patients, cannot tolerate the intensity and toxicity of standard induction chemotherapy [4,5]. Thus, new therapies are needed to improve the prognosis of patients with AML.

Leukemia initiating/stem cells are dependent on oxidative phosphorylation (OXPHOS) for energy production [6]. Standard induction chemotherapy does not eliminate OXPHOS-dependent AML cells [6,7]. Thus, targeting OXPHOS is a promising approach to improve treatment outcomes of AML patients. IACS-010759 is a selective small-molecule inhibitor of mitochondrial complex I that shows antileukemic activity in vitro as well as in AML xenograft models [8]. It is currently being investigated in Phase I clinical trials to determine tolerability, safety, and pharmacokinetics in patients with AML, relapsed refractory lymphoma, or advanced, metastatic, or unresectable solid tumors (www.clinicaltrials.gov). However, complex I deficiency has been reported to inhibit release of cytochrome c and apoptosis-inducing factor (AIF), which inhibits apoptotic cell death [9]. It is conceivable that inhibition of complex I with IACS-010759 may also block cytochrome c release, resulting in limited apoptosis in AML cells, representing a mechanism of resistance to this promising mitochondria-targeting agent.

Cytochrome c release is tightly regulated by the Bcl-2 family proteins. Antiapoptotic Bcl-2 family proteins Bcl-2, Bcl-xL, and Mcl-1 function by sequestering pro-apoptotic proteins, such as Bim, Bax, and Bak, preventing activation of Bax and/or Bak, subsequent cytochrome c release, and induction of apoptosis. The FDA-approved Bcl-2 selective inhibitor venetoclax (ABT-199) is able to disrupt the interaction between Bim and Bcl-2, freeing Bim to induce apoptosis [10,11,12,13]. Venetoclax has also been shown to target OXPHOS in AML cells in vitro [6,14]. Thus, we hypothesize that the combination of IACS-010759 and venetoclax will result in synergistic induction of intrinsic apoptosis through cytochrome c release and/or enhanced inhibition of OXPHOS in AML cells.

In this study, we investigated the antileukemic activity of venetoclax in combination with IACS-010759 in AML cell lines, primary patient samples, and an in vivo mouse model. NSGS mice were chosen for in vivo studies because they show superior engraftment of AML cell lines compared to NSG mice, making it the preferred animal model for using AML cell lines [15,16]. Our data show that venetoclax and IACS-010759 synergistically induce apoptosis in AML cell lines and primary patient samples in an OXPHOS-dependent manner. Combined IACS-010759 and venetoclax treatment significantly reduced colony formation capacity of AML progenitor cells. Further, IACS-010759 in combination with venetoclax significantly enhanced survival of an AML cell line-derived xenograft mouse model.

## 2. Results

### 2.1. IACS-010759 and Venetoclax Synergistically Induce Apoptosis in OXPHOS-Dependent AML Cell Lines

**Figure 1:**
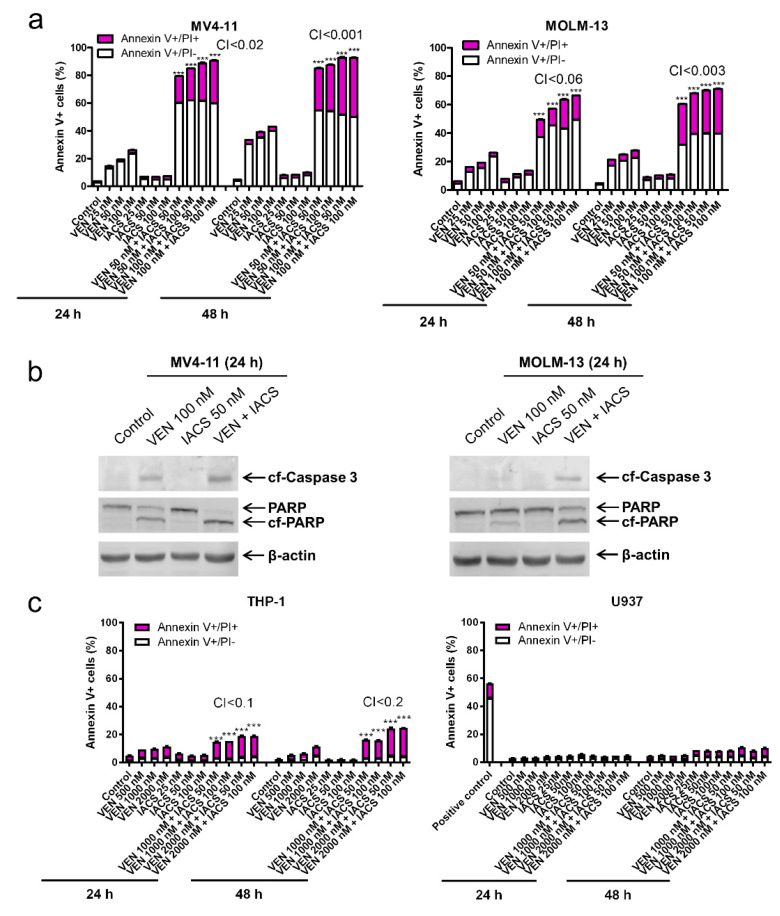

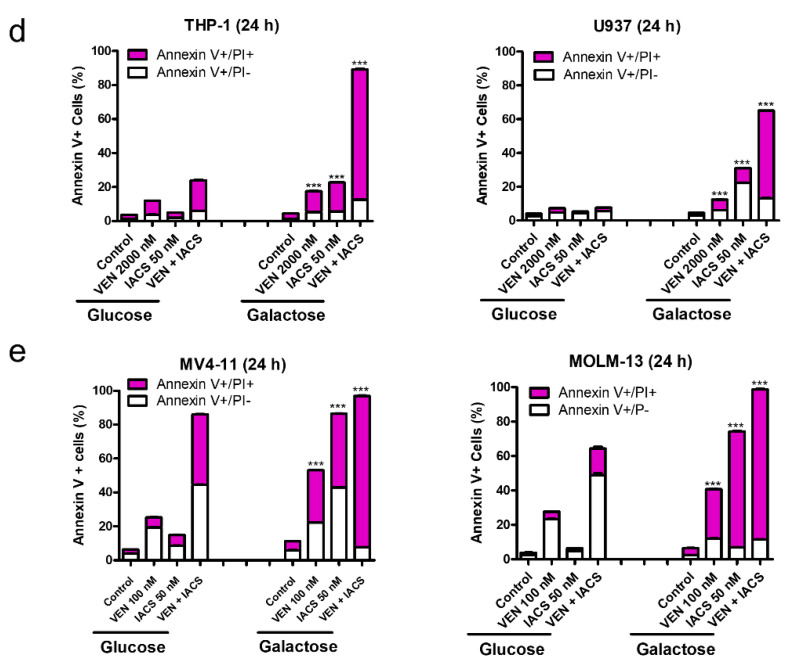
IACS-010759 synergizes with venetoclax in inducing apoptosis in oxidative phosphorylation (OXPHOS)-dependent acute myeloid leukemia (AML) cell lines. (**a**,**c**) MV4-11, MOLM-13, THP-1, and U937 cells cultured in media supplemented with glucose were treated with IACS-010759 and venetoclax alone or in combination for 24 or 48 h. The cells were stained with Annexin V-FITC/propidium iodide (PI) and analyzed by flow cytometry. Combination index (CI) values were calculated using CompuSyn software. CI < 1, CI = 1, CI > 1 indicate synergistic, additive, and antagonistic effect, respectively. *** indicates p < 0.001 when compared to vehicle control and individual drug treatments. (**b**) MV4-11 and MOLM-13 cells cultured in media supplemented with glucose were treated with IACS-010759 and venetoclax alone or in combination for 24 h. Whole cell lysates were subjected to western blotting. (**d**,**e**) THP-1, U937, MV4-11, and MOLM-13 cells were cultured in media containing glucose or galactose as the sole sugar source, in the absence or presence of IACS-010759 and venetoclax, for 12 h. Cells were stained with Annexin V-FITC/PI and analyzed by flow cytometry. *** indicates *p* < 0.001 when compared to the corresponding values in cells cultured in media supplemented with glucose.

To test our hypothesis that IACS-010759 synergizes with venetoclax in inducing apoptosis in AML cells, we tested variable concentrations of IACS-010759 and venetoclax, alone and in combination, in AML cell lines. Based on the combination index values calculated using CompuSyn software, the combination synergistically increased Annexin V positive MV4-11 and MOLM-13 cells (Figure 1a) and was accompanied by an increase in cleavage of caspase 3 and PARP (Figure 1b), both indicating induction of cellular apoptosis. U937 is an AML cell line that harbors a truncating mutation in the ND1 and ND5 subunits of complex I and are inherently more reliant on glycolysis over OXPHOS for cellular respiration [17]. Knowing that these cells have a mutation in complex I, we unsurprisingly found the combination treatment resulted in no increase of Annexin V positive U937 cells (Figure 1c), indicating lack of apoptosis induction. In THP-1 cells, we also found lower levels of Annexin V positive cells compared to the more susceptible MV4-11 and MOLM-13 cells, indicating a possible cell line difference in OXPHOS reliance for cell survival. To investigate the role of cell line reliance on OXPHOS and susceptibility to apoptosis induction by combination treatment, THP-1 and U937 cell lines were cultured in media containing only galactose as the sugar source, which forces cells to utilize OXPHOS and limits availability of glycolysis [18]. Treatment of galactose cultured THP-1 and U937 cells with IACS-010759 and venetoclax, alone and in combination, resulted in significantly increased Annexin V positive cells (Figure 1d). Similarly, treatment of galactose cultured MV4-11 and MOLM-13 cell lines with the two agents also resulted in significant further increase of Annexin V positive cells compared to glucose cultured cells (Figure 1e). Taken together, these results demonstrate that the combination treatment targets AML cells in vitro in an OXPHOS-dependent mechanism to induce cell death, and AML cell reliance on OXPHOS potentially plays an important role in sensitivity to the combination treatment.

### 2.2. IACS-010759 and Venetoclax Synergistically Induce Cell Death in Primary AML Cells and Cooperatively Reduce the Colony Forming Capacity of AML Progenitor Cells

**Figure 2:**
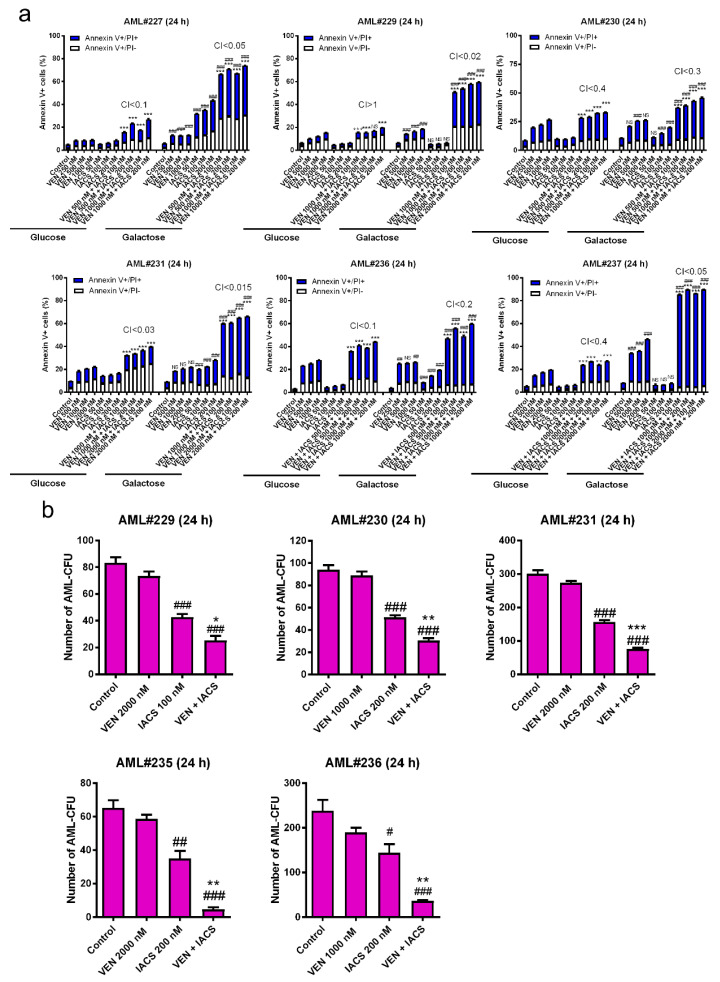
IACS-010759 and venetoclax synergistically induce cell death in primary AML cells and cooperatively reduce the colony forming capacity of AML progenitor cells. (**a**) AML patient samples (1 × 10^6^ cells/condition) were treated with IACS-010759 and venetoclax alone or in combination for 24 h in the presence of glucose or galactose as the primary sugar source. The cells were split into three tubes and then stained with Annexin V-FITC/propidium iodide (PI) and analyzed by flow cytometry. Combination index (CI) values were calculated using CompuSyn software. CI < 1, CI = 1, CI > 1 indicate synergistic, additive, and antagonistic effect, respectively. *** indicates *p* < 0.001 when compared to vehicle control and individual drug treatment, ### indicates *p* < 0.001 when compared to the corresponding values in cells cultured in media supplemented with glucose, and ns indicates not significant. (**b**) Primary AML patient samples cultured in media supplemented with glucose were treated with vehicle control, venetoclax, IACS-010759, or in combination for 24 h (1.5 × 10^6^ cells/treatment condition) and then plated in methylcellulose in triplicate (5 × 10^5^ cells/plate). After incubation for 14 days, the number of surviving AML cells capable of generating leukemia colonies (AML-CFUs) were enumerated. Data are presented as mean ± SEM. # indicates *p* < 0.05, ## indicates *p* < 0.01, and ### indicates *p* < 0.001 compared to control. * indicates *p* < 0.05, ** indicates *p* < 0.01, and *** indicates *p* < 0.001 compared to single drug treatments. Technical triplicates were performed.

To determine if OXPHOS-dependent antileukemic activity of the combination treatment also applies to primary AML cells, we compared response of primary AML cells in both glucose media and galactose media ex vivo. Similar to the cell line results, IACS-010759 and venetoclax synergistically induced variable levels of Annexin V positivity in five out of the six samples when cultured in glucose supplemented media. Analogous to the cell line models, when combination treatment was applied to the same primary samples cultured in galactose supplemented media, synergy was achieved in all six samples (Figure 2a). These results further support the notion that the combination of IACS-010759 and venetoclax target AML cells to induce cell death through an OXPHOS-dependent manner.

To determine the effect of IACS-010759 and venetoclax on leukemic progenitor cells, colony-forming assays were performed using primary AML patient samples (*n* = 5). Venetoclax alone did not have a significant impact on colony forming capacity (Figure 2b). In contrast, IACS-010759 significantly decreased colony formation, which was further and significantly decreased by combination with venetoclax (Figure 2b), demonstrating that this combination treatment has activity against leukemic progenitor cells in vitro.

### 2.3. Venetoclax Significantly Enhances the Antileukemic Activity of IACS-010759 in an AML Cell Line-Derived Xenograft Mouse Model

**Figure 3:**
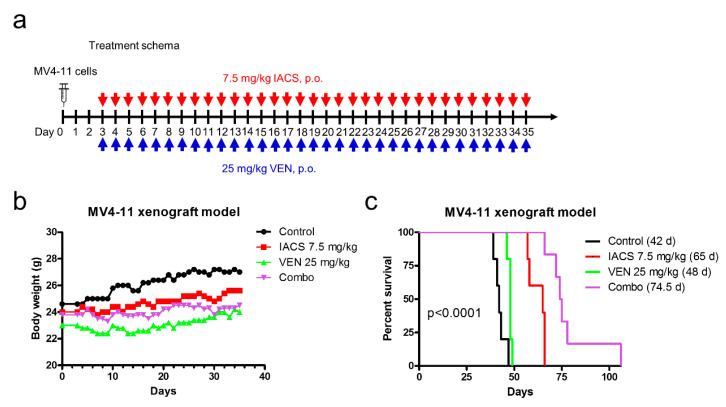
IACS-010759 and venetoclax combination shows in vivo efficacy in an AML cell line-derived xenograft mouse model. (**a**) NSGS mice were injected with MV4-11 cells (1 × 10^6^ cells/mouse, via tail vein injection). Three days post cell injection the mice were randomized and treated. Vehicle control, 7.5 mg/kg IACS-010759, 25 mg/kg venetoclax, or 7.5 mg/kg IACS-010759 + 25 mg/kg venetoclax (*n* = 5) were administered on a daily basis via oral gavage for a total of 33 days. (**b**,**c**) Average mouse body weights were measured on a daily basis and graphed (panel b). Overall survival probability, estimated with the Kaplan-Meier method, is shown in panel c.

To determine in vivo efficacy of IACS-010759 in combination with venetoclax, we evaluated the combination in a MV4-11-derived xenograft mouse model. Starting day three post cell injection, mice were treated daily for thirty-three days (until the appearance of leukemia symptoms) with either vehicle control, IACS-010759 at 7.5 mg/kg p.o., venetoclax at 25 mg/kg p.o., or the combination of IACS-010759 and venetoclax (5 mice/group; Figure 3a). Body weight loss of 3% or less was observed during treatment (Figure 3b), demonstrating that the combination was well tolerated. Every single mouse died from AML symptoms (hindleg weakness, >15% weight loss, metastatic spread to internal organs). Median survival for vehicle control, IACS-010759, venetoclax, and the combination were 42 days, 65 days, 48 days, and 74.5 days (a 77.4% increase in life span compared to vehicle control), respectively (Figure 3c). These results demonstrate that the combination of IACS-010759 and venetoclax is well tolerated and has promising antileukemic activity in vivo.

### 2.4. Inhibition of OXPHOS by IACS-010759 Treatment is not Enhanced by Venetoclax

**Figure 4:**
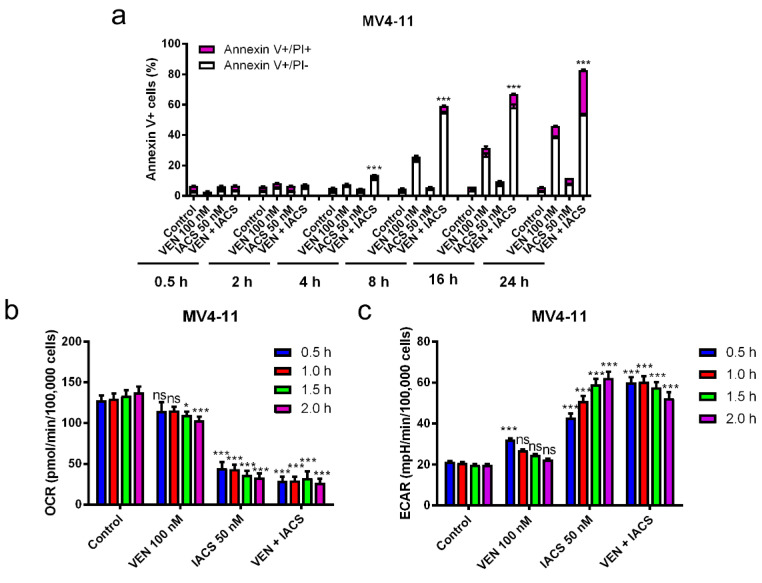

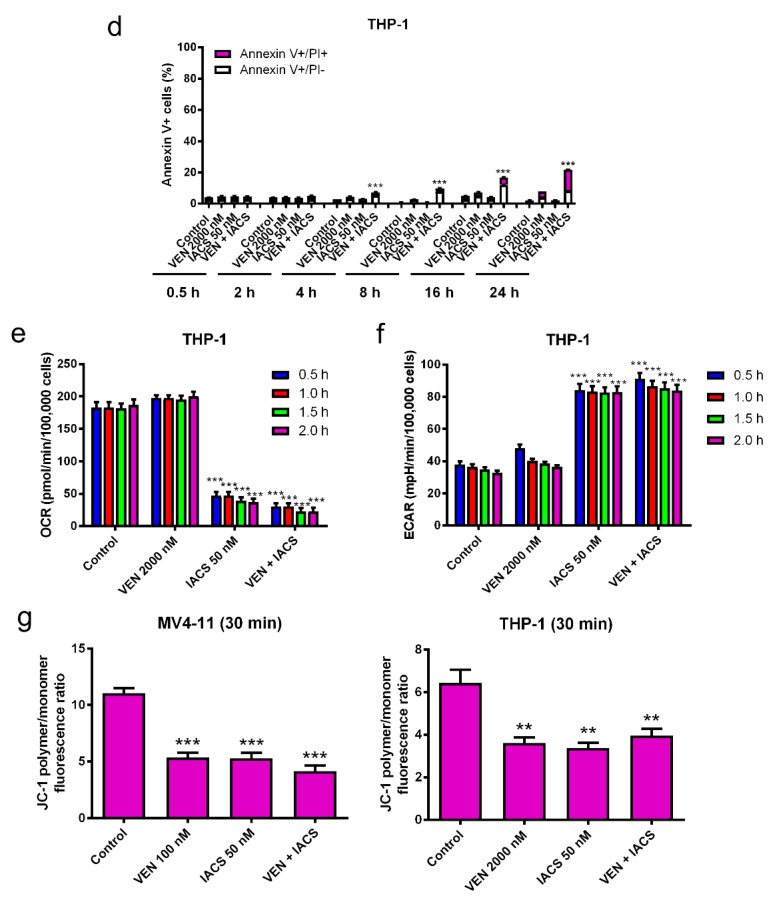
Inhibition of OXPHOS by IACS-010759 treatment is not enhanced by venetoclax. (**a**,**d**) MV4-11 and THP-1 cells cultured in media supplemented with glucose were treated with IACS-010759 and venetoclax alone and in combination for up to 24 h. Cells were then stained with Annexin V/PI and analyzed by flow cytometry. ** indicates *p* < 0.01 and *** indicates *p* < 0.001 compared to vehicle control and single drug treatments. (**b**,**c**,**e**,**f**) MV4-11 and THP-1 cells cultured in media supplemented with glucose were plated in Seahorse media and incubated in a Seahorse analyzer. IACS-010759 and venetoclax were injected into the cell culture while in the Seahorse analyzer. Oxygen consumption rate (OCR) and extracellular acidification rate (ECAR) were measured every 30 min for up to 2 h after injection of the drugs. * indicates *p* < 0.05, *** indicates *p* < 0.001, and ns indicates not significant compared to vehicle control. (**g**) MV4-11 and THP-1 cells cultured in media supplemented with glucose were treated with vehicle control, venetoclax, IACS-010759, or in combination for 30 min and then the cells were subjected to the JC-1 assay. ** indicates *p* < 0.01 and *** indicates *p* < 0.001 compared to vehicle control.

Since inhibition of Bcl-2 has been shown to inhibit OXPHOS [6], we next sought to determine if venetoclax enhances IACS-010759-induced decrease of OXPHOS. First, we treated MV4-11 cells with IACS-010759 and venetoclax to determine when significantly increased Annexin V positive cells could be detected. At 4 h post combination treatment, we detected significantly increased Annexin V positive cells, and no increase of Annexin V positive cells could be detected at 2 h post combination treatment (Figure 4a). Based on this finding, MV4-11 cells were treated with IACS-010759 and venetoclax alone and in combination for up to 2 h and the oxygen consumption rate (OCR) and extracellular acidification rate (ECAR) were monitored via Seahorse extracellular flux analyzer (Seahorse Biosicence). Consistent with the reported OXPHOS inhibitor activity [8], IACS-010759 treatment of MV4-11 cells significantly reduced OCR (Figure 4b). This was accompanied by significant increase of ECAR (Figure 4c). In contrast, venetoclax had little to no effect on OCR and ECAR, whereas the combination treatment resulted in OCR and ECAR changes similar to that seen in IACS-010759 treatment alone. Similar results were also obtained in THP-1 cells (Figure 4d–f). While both IACS-010759 and venetoclax treatment alone were able to reduce mitochondrial membrane potential (MMP), no enhancement was seen in the combination treatment (Figure 4g). Taken together, these results suggest that while IACS-010759 treatment alone reduces OCR, increases ECAR, and reduces MMP, these changes are not enhanced by venetoclax and do not explain how venetoclax enhances antileukemic activity of IACS-010759.

### 2.5. IACS-010759 Induces a Vulnerable Mitochondrial State that Sensitizes AML Cells to Venetoclax Induced Cytochrome c Release

**Figure 5:**
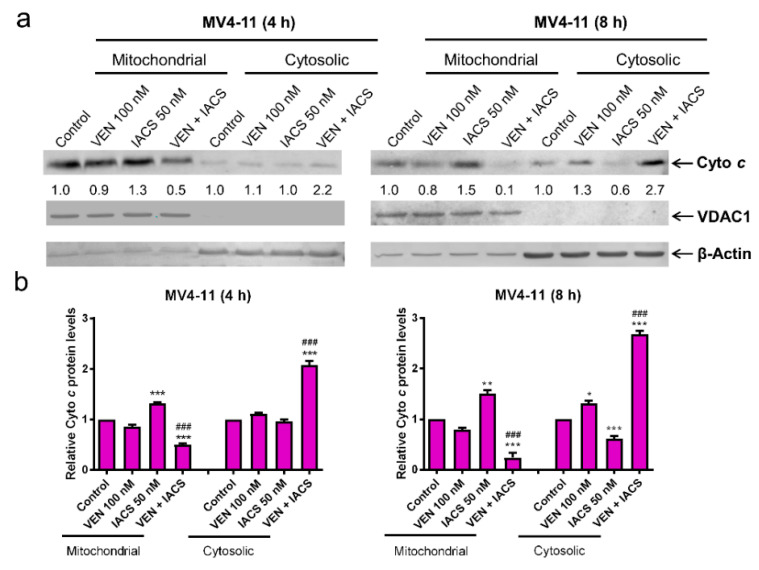

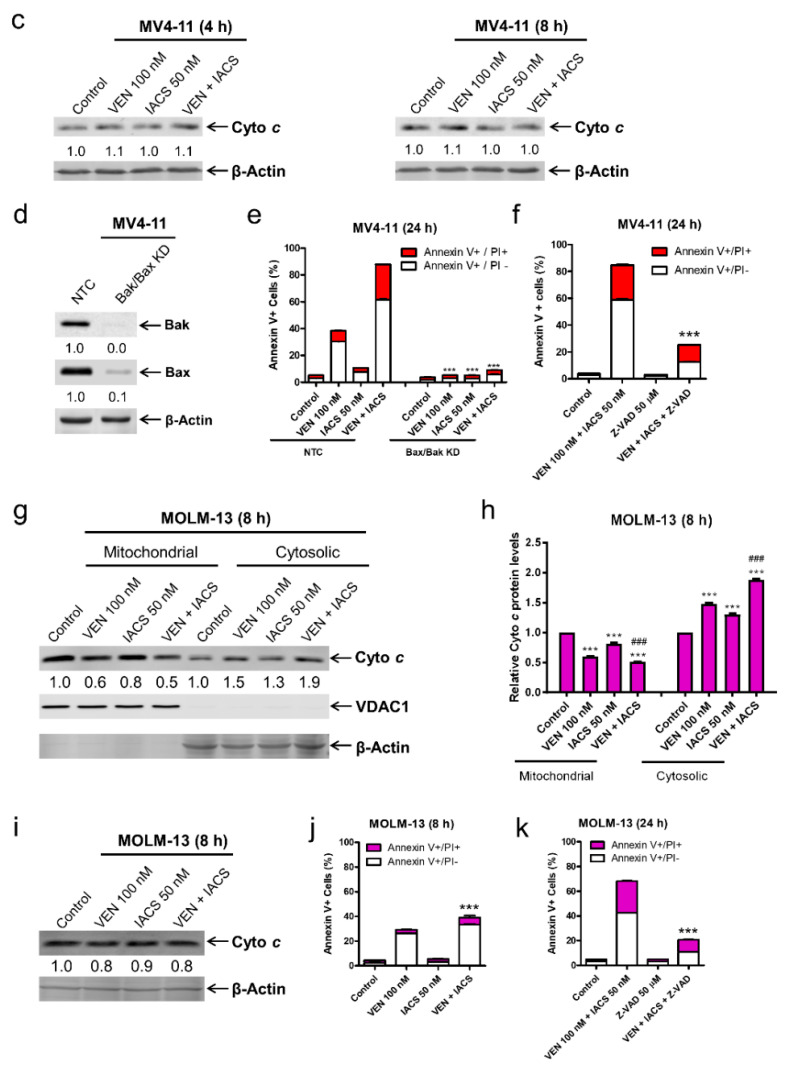
IACS-010759 and venetoclax cooperatively induce intrinsic apoptosis. (**a**,**b**) MV4-11 cells cultured in media supplemented with glucose were treated with vehicle control, IACS-010759, venetoclax, or in combination for 4 or 8 h. Cellular fractionation was performed. Mitochondrial and cytosolic fractions were subjected to western blot analysis. This experiment was performed 2 independent times in triplicate. One representative image is shown. Relative densitometry measurements were determined using Odyssey Software V3.0, normalized to β-actin or VDAC1, and compared to the vehicle control. Results from one representative experiment are graphed as mean ± SEM in panel b. * indicates *p* < 0.05, ** indicates *p* < 0.01, and *** indicates *p* < 0.001 compared to vehicle control; ### indicates *p* < 0.001 compared to single drug treatments. (**c**) MV4-11 cells were cultured and treated as described in panel a. Whole cell lysates were subjected to western blot analysis. Relative densitometry measurements were determined using Odyssey Software V3.0, normalized to β-actin, and compared to the vehicle control. (**d**,**e**) Lentiviral shRNA double knockdown of Bak and Bax (designated Bak/Bax KD) was performed in MV4-11 cells. Non-template control (NTC)-shRNA was used as the control for the Bak/Bax double knockdown. Western blots probed with anti-Bak, -Bax, or -β-actin antibody are shown in panel d. shRNA knockdown cells cultured in media supplemented with glucose were treated with IACS-010759 and venetoclax, alone or combined, for 24 h. Cells were then stained with Annexin V-FITC/PI and analyzed by flow cytometry. *** indicates *p* < 0.001 compared to the NTC-shRNA cells. (**f**) MV4-11 cells cultured in media supplemented with glucose were treated with combined IACS-010759 and venetoclax in the absence or presence of the pan-caspase inhibitor Z-VAD-FMK for 24 h. Cells were then stained with Annexin V-FITC/PI and analyzed by flow cytometry. *** indicates *p* < 0.001 compared to the combined venetoclax and IACS-010759 treatment. (**g**,**h**) MOLM-13 cells cultured in media supplemented with glucose were treated with vehicle control, IACS-010759, venetoclax, or in combination for 8 h. (**i**) Cellular fractionation was performed. Mitochondrial and cytosolic fractions were subjected to western blot analysis. This experiment was performed 2 independent times in triplicate. One representative image is shown. Relative densitometry measurements were determined using Odyssey Software V3.0, normalized to β-actin or VDAC1, and compared to the vehicle control. Results from one representative experiment are graphed as mean ± SEM in panel h. *** indicates *p* < 0.001 compared to vehicle control; ### indicates *p* < 0.001 compared to single drug treatments. (**j**) MOLM-13 cells cultured in media supplemented with glucose were treated with IACS-010759 and venetoclax, alone or combined, for 8 h. Cells were then stained with Annexin V-FITC/PI and analyzed by flow cytometry. *** indicates *p* < 0.001 compared to the vehicle control and single drug treatments. (**k**) MOLM-13 cells cultured in media supplemented with glucose were treated with combined IACS-010759 and venetoclax in the absence or presence of the pan-caspase inhibitor Z-VAD-FMK for 24 h. Cells were then stained with Annexin V-FITC/PI and analyzed by flow cytometry. *** indicates *p* < 0.001 compared to the combined venetoclax and IACS-010759 treatment.

The lack of enhancement on IACS-010759-inudced inhibition of OCR by venetoclax prompted us to consider the possibility that venetoclax enhances the antileukemic activity through release of IACS-010759 induced sequestration of cytochrome c. To test this possibility, OXPHOS-reliant MV4-11 cells were treated with the drug combination for 4 or 8 h and western blot analysis was used to determine relative changes in cytochrome c localization. Consistent with our hypothesis that inhibition of OXPHOS complex I retains cytochrome c in the mitochondria, IACS-010759 treatment of MV4-11 cells for 4 or 8 h resulted in a significant increase in cytochrome c within the mitochondrial fraction (Figure 5a,b). Venetoclax treatment alone resulted in low levels (~30% at the 8 h timepoint) of cytochrome c release from MV4-11 cells, but when used in combination it promoted a greater than 2-fold increase in cytochrome c release from the mitochondria (Figure 5a,b). Total levels of cytochrome c remained unchanged between IACS-010759 and control, suggesting that higher levels of cytochrome c in the mitochondria were not due to induction of protein synthesis (Figure 5c). Similar results were obtained in another OXPHOS-reliant AML cell line MOLM-13 (Figure 5g–j). These results indicate that venetoclax enhances IACS-010759-induced apoptosis through enhancement of cytochrome c release. To confirm that cytochrome c release is the main cause of apoptosis induced by IACS-010759 and venetoclax, the intrinsic apoptosis pathway was blocked via a double knockdown of Bak and Bax in MV4-11 cells (Bak/Bax KD, Figure 5d). This resulted in an almost complete rescue of the Bak/Bax KD cells from combined treatment (Figure 5d,e). To provide additional evidence regarding the role of cytochrome c release in apoptosis induced by the two agents, MV4-11 cells were treated with the pan-caspase inhibitor Z-VAD-FMK in the presence or absence of combined IACS-010759 and venetoclax. Z-VAD-FMK almost completely rescued the cells from apoptosis induced by the combination (Figure 5f). Similar results were obtained in MOLM-13 cells (Figure 5k).

**Figure 6:**
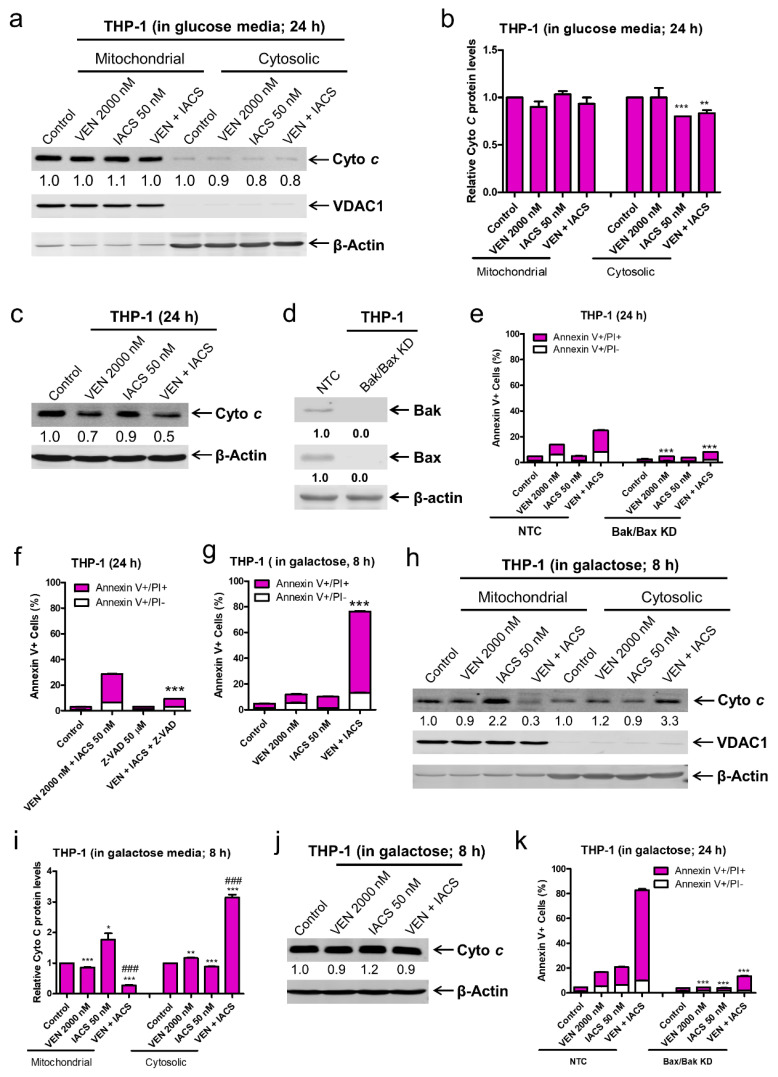
Forcing glycolysis-reliant AML cells to utilize OXPHOS renders them susceptibility to IACS-010759 and venetoclax induced intrinsic apoptosis mediated by cytochrome c release. (**a**,**b**) THP-1 cells, cultured in media supplemented with glucose, were treated with vehicle control, IACS-010759, venetoclax, or in combination for 24 h. Cellular fractionation was performed. Mitochondrial and cytosolic fractions were subjected to Western blot analysis. This experiment was performed 2 independent times in triplicate. One representative image is shown. Relative densitometry measurements were determined using Odyssey Software V3.0, normalized to β-actin or VDAC1, and compared to the vehicle control. Results from one representative experiment are graphed as mean ± SEM in panel b. * indicates *p* < 0.05, ** indicates *p* < 0.01, and *** indicates *p* < 0.001 compared to vehicle control. (**c**) THP-1 cells were cultured and treated as described in panel a. Whole cell lysates were subjected to Western blot analysis. Relative densitometry measurements were determined using Odyssey Software V3.0, normalized to β-actin, and compared to the vehicle control. (**d**,**e**) Lentiviral shRNA double knockdown of Bak and Bax (designated Bak/Bax KD) was performed in THP-1 cells. Non-template control (NTC)-shRNA was used as the control for the Bak/Bax double knockdown. Western blots probed with anti-Bak, -Bax, or -β-actin antibody are shown in panel d. shRNA knockdown cells cultured in media supplemented with glucose were treated with IACS-010759 and venetoclax, alone or combined, for 24 h. Cells were then stained with Annexin V-FITC/PI and analyzed by flow cytometry. *** indicates *p* < 0.001 compared to the NTC-shRNA cells. (**f**) THP-1 cells cultured in media supplemented with glucose were treated with combined IACS-010759 and venetoclax in the absence or presence of the pan-caspase inhibitor Z-VAD-FMK for 24 h. Cells were then stained with Annexin V-FITC/PI and analyzed by flow cytometry. *** indicates *p* < 0.001 compared to the combined venetoclax and IACS-010759 treatment. (**g**–**i**) THP-1 cells cultured in media supplemented with galactose were treated with vehicle control, IACS-010759, venetoclax, or in combination for 8 h. In panel g, cells were then stained with Annexin V-FITC/PI and analyzed by flow cytometry. *** indicates *p* < 0.001 compared to the vehicle control and single drug treatments. For panels (**h**,**i**), cellular fractionation was performed. Mitochondrial and cytosolic fractions were subjected to Western blot analysis. This experiment was performed 2 independent times in triplicate. One representative image is shown. Relative densitometry measurements were determined using Odyssey Software V3.0, normalized to β-actin or VDAC1, and compared to the vehicle control. Results from one representative experiment are graphed as mean ± SEM in panel **i**. * indicates *p* < 0.05, ** indicates *p* < 0.01, and *** indicates *p* < 0.001 compared to vehicle control, ### indicates *p* < 0.001. (**j**) THP-1 cells were cultured and treated as described in panel (**g**). Whole cell lysates were subjected to Western blot analysis. Relative densitometry measurements were determined using Odyssey Software V3.0, normalized to β-actin, and compared to the vehicle control. (**k**) THP-1 NTC and Bak/Bax double knockdown cells cultured in media supplemented with galactose were treated with IACS-010759 and venetoclax, alone or combined, for 24 h. Cells were then stained with Annexin V-FITC/PI and analyzed by flow cytometry. *** indicates *p* < 0.001 compared to the NTC-shRNA cells.

To further establish the mechanism underlying the synergistic induction of intrinsic apoptosis in AML cells by IACS-010759 and venetoclax, cytochrome c release was measured in the more glycolysis-reliant cell line THP-1 cultured either in glucose or galactose supplemented media post treatment with the two agents, alone or combined. THP-1 cells cultured in glucose media showed little (~30%) to no induction of apoptosis as well as no significant increase in cytochrome c release (Figure 6a,b). Total levels of cytochrome c were decreased in combination treated cells (Figure 6c). Bak/Bax double knockdown and the pan-caspase inhibitor Z-VAD-FMK almost completely rescued of the cells from combined treatment (Figure 6d–f). Forcing the THP-1 cells to utilize OXPHOS by changing the sugar source from glucose to galactose resulted in significant induction of apoptosis (~75%) and cytochrome c release after just 8 h of combined drug treatment (Figure 6g–i), while total levels of cytochrome *c* remained largely unchanged (Figure 6j). In galactose media, double knockdown of Bak and Bax almost completely rescued THP-1 cells from combined drug treatment (Figure 6k). These data show that forcing glycolysis-reliant cells to mainly utilize OXPHOS enhances cytochrome c release and intrinsic apoptosis induced by combined IACS-010759 and venetoclax. Taken together, these results demonstrate that IACS-010759 treatment retains cytochrome c in the mitochondria, which is abolished by venetoclax and leads to intrinsic apoptosis in OXPHOS-reliant AML cells.

## 3. Discussion

Our findings demonstrate that IACS-010759 and venetoclax synergistically induce apoptosis in OXPHOS-reliant AML cells. Recently it was reported that venetoclax suppresses mitochondrial respiration through inhibition of complex I [19] and complex II (when used in combination with azacitidine) [14]. Interestingly, our results suggest that while inhibition of OXPHOS plays a role in IACS-010759 activity, cooperative inhibition of OXPHOS is not likely responsible for the synergistic antileukemic activity of combined IACS-010759 and venetoclax (Figure 4), though it cannot be ruled out.

Complex I deficiency has been reported to inhibit release of cytochrome c and apoptosis-inducing factor, which inhibits apoptotic cell death [9]. In line with this, our data shows that inhibition of complex I via IACS-010759 retains cytochrome c in the mitochondrial fraction (Figure 5). Inhibition of Bcl-2 via venetoclax decreases cytochrome c in the mitochondrial fraction and increases it in the cytosolic fraction, though only to a small degree when used alone (Figure 5). However, when venetoclax was combined with IACS-010759, it completely abolished the sequestration of cytochrome c induced by IACS-010759, leading to an increase in cytochrome c in the cytosolic fraction, suggesting that this is the main mechanism underlying the synergy between the two agents in OXPHOS-reliant AML cells (Figure 5). The role of cytochrome c release in the synergistic induction of apoptosis by the two agents was supported by the significant reduction of apoptosis seen in both Bak/Bax double knockdown cells and cells treated with a pan-caspase inhibitor (Figure 5e,f and Figure 6k). It is noted that the reliance of apoptosis on Bak/Bax and caspases in the combination treatment does not appear to be complete, as there was some increase in apoptosis in the combination treatment compared to control despite double knockdown of Bak/Bax and caspase inhibition. The degree of Bak/Bax knockdown and the concentration of the pan-caspase inhibitor were found to be sufficient to completely rescue these cells from treatment with venetoclax and other intrinsic apoptosis inducers (Figure 5e). This suggests that a Bak/Bax-independent and/or caspase-independent mechanism may play a small role in cell death induction by the combination of IACS-010759 and venetoclax. It is also important to note that the lack of cytochrome c release in THP-1 cells cultured in glucose supplemented media post combined IACS-010759 and venetoclax treatment may simply be due to the sensitivity of the assay. However, we cannot rule out the possibility that IACS-010759 and venetoclax cooperate in inducing release of other proteins in the mitochondrial intermembrane space, leading to Bak/Bax-dependent apoptosis. Furthermore, IACS-010759 and venetoclax could cooperate in inhibiting AML cell proliferation, though its role in tumor growth inhibition would be minor since combined treatment of MV4-11 cells cultured in glucose supplemented media and THP-1 cells cultured in galactose supplemented media (both cell lines are OXPHOS-dependent under these experimental conditions) for 24 h resulted in 90% cell death, accompanied by significantly increased cytochrome c release. Additional studies are warranted, however they are not in the scope of this paper.

Consistent with the findings of Molina and colleagues [8], we found that IACS-010759 treatment prolonged survival of an early stage AML cell line-derived xenograft mouse model. Additionally, venetoclax further increased the median survival of mice treated with IACS-010759 (Figure 3), demonstrating that the combination of IACS-010759 and venetoclax shows enhanced antileukemic activity in vivo. Additionally, similar to conclusions drawn by Molina and colleagues, we found evidence that the combination therapy is more selective for AML cells that rely more heavily on OXPHOS. This was evident by both the increased susceptibility to cell death under glycolysis inhibition circumstances (galactose only media) and by a quick and maximal switch to glycolysis in more IACS-010759 resistant cell lines, as evident by the ECAR response that is seen in THP-1 cells compared to MV4-11 cells (Figure 4). Based on the reported reliance of leukemia initiating/stem cells on OXPHOS for survival [6], this combination might have promising activity against leukemia initiating/stem cells, which was preliminarily demonstrated here through the colony formation assay results, which show that the combination has antileukemic activity against AML progenitor cells (Figure 2b).

As expected, IACS-010759 treatment did not induce Annexin V positivity in U937 cells, which harbor a truncating mutation in the ND1 and ND5 subunits of OXPHOS complex I [17]. This inherent cellular reliance on glycolysis could be overcome by forcing U937 cells to use galactose as the sole sugar source, forcing the cells to utilize OXPHOS. This treatment demonstrated a significant increase in Annexin V positive cells following IACS-010759 treatment alone, which further increased upon combined treatment with IACS-010759 and venetoclax (Figure 1d). We speculate that U937 cells retain a minimal level of complex I activity such that under these experimental conditions the cells respond to complex I inhibition. However, further studies to rule out off-target effects of IACS-010759 are warranted.

## 4. Materials and Methods

### 4.1. Drugs

IACS-010759 and venetoclax were purchased from Selleck Chemicals (Houston, TX, USA). Z-VAD-FMK was purchased from Sigma-Aldrich (St. Louis, MO, USA).

### 4.2. Cell Lines

MV4-11, U937, and THP-1 were purchased from the American Type Culture Collection (Manassas, VA, USA). MOLM-13 was purchased from AddexBio (San Diego, CA, USA). The cell lines were cultured as previously described [20]. The cell lines were authenticated in August 2017 at the Genomics Core at Karmanos Cancer Institute using the PowerPlex^®^ 16 System from Promega (Madison, WI, USA). The cell lines were tested for the presence of mycoplasma by PCR on a monthly basis [21].

### 4.3. Clinical Samples

Diagnostic blast samples were obtained from the First Hospital of Jilin University, Changchun, China. Written informed consent was provided according to the Declaration of Helsinki. This study was approved by the Human Ethics Committee of The First Hospital of Jilin University (Ethical code #: 2019-128). Clinical samples were screened for FLT3-ITD, NPM1, C-kit, CEBPA, IDH1, IDH2, and DNMT3A gene mutations by PCR amplification and automated DNA sequencing and for fusion genes by real-time RT-PCR, as described previously [22,23]. Patient characteristics are shown in Table 1.

### 4.4. Annexin V/PI Staining and Flow Cytometry Analysis

Following drug treatment, AML cells were stained with Annexin V-fluorescein isothiocyanate (FITC)/propidium iodide (PI) (Beckman Coulter, Brea, CA, USA) and then subjected to flow cytometry analysis, as previously described [24,25]. Apoptotic events are displayed as mean percentage of Annexin V+/PI− (early apoptotic) and Annexin V+/PI+ (late apoptotic and/or dead) cells ± the standard error from one representative experiment. Combination index values (CI) were calculated using CompuSyn software (ComboSyn Inc. Paramus, NJ, USA). CI < 1, CI = 1, CI > 1 indicate synergistic, additive, and antagonistic effects, respectively.

### 4.5. Western Blotting

Western blotting was performed as previously described [24]. Antibodies directed towards β-actin, Bax, PARP (Proteintech, Chicago, IL, USA), cleaved (cf) caspase 3, Bim (Cell Signaling Technologies, Danvers, MA, USA), Bak (Abcam, Cambridge, MA, USA), cytochrome c, and VDAC1 (Biomake.cn, Shanghai, China) were used for western blot analysis (uncropped western blot can be found at Figure S1).

### 4.6. Assessment of Mitochondrial Membrane Potential

AML cells were treated with IACS-010759 and venetoclax, alone or combined, for 30 min and then resuspended in fresh media containing 1 μM JC-1 (Solarbio, Beijing, China) and incubated for 15 min at 37 °C. The samples were washed, resuspended in PBS, and then assessed by flow cytometry analyses.

### 4.7. Mitochondrial Fractionation/Cytochrome c Release

Mitochondria were isolated using the Mitochondria Extraction Kit (Solarbio Science and Technology, Beijing, China), according to the manufacturer’s instructions. Briefly, 5 × 10^7^ cells were collected after drug treatment, washed with 10 mL of ice-cold PBS, and centrifuged at 800 × *g* for 5 min at 4 °C. The supernatant was discarded, and cells were resuspended in 1 mL of cold Lysis buffer. Cells were homogenized using a Dounce tissue grinder for 30–40 strokes on ice. The homogenate was centrifuged at 1000 × *g* for 5 min at 4 °C. The supernatant was transferred to a fresh 1.5 mL tube, centrifuged at 12,000 × *g* for 10 min at 4 °C, and the supernatant was collected as the cytosolic fraction, while the pellet (mitochondrial fraction) was resuspended with 500 µL Wash Buffer, centrifuged at 1000 × *g* for 5 min at 4 °C. The supernatant was transferred to a 1.5 mL tube, centrifuged at 12,000 × *g* for 10 min at 4 °C. After centrifugation, the supernatant was discarded and the pellet (mitochondrial fraction) was resuspended with 50–100 µL of Storage Buffer.

### 4.8. Leukemia Xenograft Model

Eight-week old immunocompromised triple transgenic NSG-SGM3 female mice (NSGS, JAX#103062; non-obese diabetic scid gamma (NOD.Cg-Prkdc^scid^ Il2rg^tm1Wjl^ Tg(CMV-IL3, CSF2, KITLG)1Eav/MloySzJ; Jackson Laboratory, Bar Harbor, ME, USA) were injected intravenously with MV4-11 cells (1 × 10^6^ cells/mouse; 0.2 mL/inj.; day 0). On day 3, mice were randomized (5 mice/group; this was based on traditional design and cost of the study) into vehicle control [3% ethanol (200 proof), 1% Tween-80 (polyoxyethylene (20) sorbitan monooleate), and sterile water; all USP grade], 7.5 mg/kg IACS-010759, 25 mg/kg venetoclax, and 7.5 mg/kg IACS-010759 + 25 mg/kg venetoclax. Mice were treated orally, on a daily basis, in the morning. Vehicle control and venetoclax were administered first, followed by IACS-010759 2 h later. Body weight and condition were assessed 1–2 times a day for the duration of study. Experimental endpoint and efficacy response were determined for each group based on the median day for development of leukemic symptoms (hindleg weakness, >15% weight loss, metastatic spread to internal organs). All mice were provided food (5021, LabDiet, St. Louis, MO, USA) and water ad libitum, given supportive fluids and supplements as needed, and housed within an AAALAC accredited animal facility with 24/7 veterinary care. Animal housing rooms are under temperature, light, and humidity control. The animals were housed, 5–6 mice per cage, in large cages fitted with micro filters and filled with Bed-o’Cobs (laboratory animal bedding). All mice were euthanized by cervical dislocation under isoflurane anesthesia. In vivo experiments were approved by the Institutional Animal Care and Use Committee at Wayne State University (IACUC# 17-08-315).

### 4.9. shRNA Knockdown

Lentivirus production and transduction were carried out, as previously described [26]. The pMD-VSV-G and delta 8.2 plasmids were gifts from Dr. Dong at Tulane University. Non-template negative control (NTC)-, Bax-, and Bak-shRNA lentiviral constructs were purchased from Sigma-Aldrich (St. Louis, MO, USA).

### 4.10. Measurement of Oxygen Consumption Rate and Glycolysis

All Seahorse assays were performed in an XF^e^24 analyzer (Seahorse Bioscience, North Billerica, MA, USA). XF24 cell culture microplates (#100777-004, Agilent Technologies, Woburn, MA, USA) were coated with 50 µL Cell-Tak adhesive solution (#354240, Corning, Glendale, AZ, USA) as previously described [27]. MV4-11 and THP-1 cells were seeded at a density of 100,000 cells/well in a total volume of 150 µL of DMEM supplemented with 11 mM glucose, 1 mM sodium pyruvate, and 2 mM glutamine. The microplates were spun down at 100 *g* for 1 min to settle the cells, 425 µL of supplemented DMEM was carefully added to each well and the cells were incubated for 1 h at 37 °C in a CO2-free incubator. During the course of the experiment, venetoclax (100 nM, 2 µM), IACS-010759 (50 nM), and the combination of the two drugs were injected into the microplates. OCR, as an indicator of mitochondrial respiration, and ECAR, as an indicator of glycolysis, were measured every 30 min for 2 h following the addition of the drugs.

### 4.11. Colony Formation Assay

Colony formation assays were performed as previously described [20]. Briefly, primary AML cells were treated with venetoclax and IACS-010759, alone or in combination for 24 h, washed with PBS, plated in triplicate in MethoCult (catalog number 04434; Stem Cell Technologies, Cambridge, MA, USA), and incubated for 14 days, according to the manufacturer’s instructions. Colony forming units were visualized using an inverted microscope and colonies with >50 cells were enumerated.

### 4.12. Statistical Analysis

Differences in cellular apoptosis (comparison of the sum of Annexin V+/PI− and Annexin V+/PI+ cells) between treatment groups and/or untreated cells were compared by pairwise two-sample t-test. One-way ANOVA was used to compare differences between three or more groups with Bonferroni correction. Overall survival probability was estimated (Kaplan-Meier method) and statistical analysis was performed using the log-rank test. Statistical analyses were performed utilizing GraphPad Prism 5.0. Error bars represent ± standard error of the mean (s.e.m.); significance level was set at *p* < 0.05.

## 5. Conclusions

In summary, our work shows that the combination of IACS-010759 and venetoclax has promising antileukemic activity against OXPHOS-reliant AML cells. Since AML initiating/stem cells are reliant on OXPHOS for survival, this combination will likely show efficacy against AML initiating/stem cells. Our study supports further investigation of the combination and the potential for clinical translation.

## Figures and Tables

**Table 1:** Patient characteristics of primary AML patient samples.

Patient	Gender	Age (Year)	Disease Status	CYTOGENETICS	Blast Purity (%)	Gene Mutation
AML#227	Male	32	Newly diagnosed	47, XY,+14, inv(16)(p13q22)/46, XY, inv(16)(p13q22)/46, XY	87.5	PRAME, WT1, JAK2, NRAS
AML#229	Male	9	Newly diagnosed	46, XY, t(15,17)(q24;q21)/46, XY	92.0	PML-RARαL, EP300, KRAS
AML#230	Female	16	Newly diagnosed	46, XX, t(8;21)(q22;q22)/46, XX	59.0	AML-ETO, WT1, PRAME
AML#231	Male	17	Newly diagnosed	46, XY, der(19)t(1;19)(q23;p13), + 21	74.0	ND
AML#235	Female	33	Newly diagnosed	46, XX	87.0	NPM1
AML#236	Male	26	Newly diagnosed	46, XY, inv(17)(q10), t(15;17)(q24;21)	88	WT1, PRAME, PML-RARα(+)
AML#237	Male	56	Relapsed	46, XY	88.0	NA

ND: not detected; NA: not available.

## Supplementary Materials

The following are available online at https://www.mdpi.com/2072-6694/12/9/2400/s1, Figure S1: Uncropped Western Blot.
