# Supplementary material for: Cotargeting of Mitochondrial Complex I and Bcl-2 Shows Antileukemic Activity against Acute Myeloid Leukemia Cells Reliant on Oxidative Phosphorylation
Source: Cancers (Basel). 2020 Aug 24;12(9):2400. doi: 10.3390/cancers12092400 (PMC7564145; doi:10.3390/cancers12092400)
Supplement: Supplementary file 1 [file cancers-12-02400-s001.pdf]

Cotargeting of Mitochondrial Complex I and Bcl-2 Shows Antileukemic Activity against Acute Myeloid Leukemia Cells Reliant on Oxidative Phosphorylation

Fangbing Liu [1], Hasini A. Kalpage [2], Deying Wang [3], Holly Edwards [4,5], Maik Hüttemann [2], Jun Ma [1], Yongwei Su [1,4,5], Jenna Carter [6], Xinyu Li [1], Lisa Polin [4,5], Juiwanna Kushner [4,5], Sijana H Dzinic [4,5], Kathryn White [4,5], Guan Wang [1*], Jeffrey W. Taub [7,8*], and Yubin Ge [4,5,6*].

[1] National Engineering Laboratory for AIDS Vaccine, Key Laboratory for Molecular Enzymology and Engineering, the Ministry of Education, School of Life Sciences, Jilin University, Changchun, China
[2] Center for Molecular Medicine and Genetics, Wayne State University School of Medicine, Detroit, MI
[3] The Tumor Center of the First Hospital of Jilin University, Changchun, P. R. China
[4] Department of Oncology, Wayne State University School of Medicine, Detroit, MI
[5] Molecular Therapeutics Program, Barbara Ann Karmanos Cancer Institute, Wayne State University School of Medicine, Detroit, MI
[6] Cancer Biology Graduate Program, Wayne State University School of Medicine, Detroit, MI
[7] Division of Pediatric Hematology/Oncology, Children's Hospital of Michigan, Detroit, MI
[8] Department of Pediatrics, Wayne State University School of Medicine, Detroit, MI

* Correspondence: gey@karmanos.org (Y.G.); jtaub@med.wayne.edu (J.W.T.); wg10@jlu.edu (W.G.).

Fig. 1 A

a

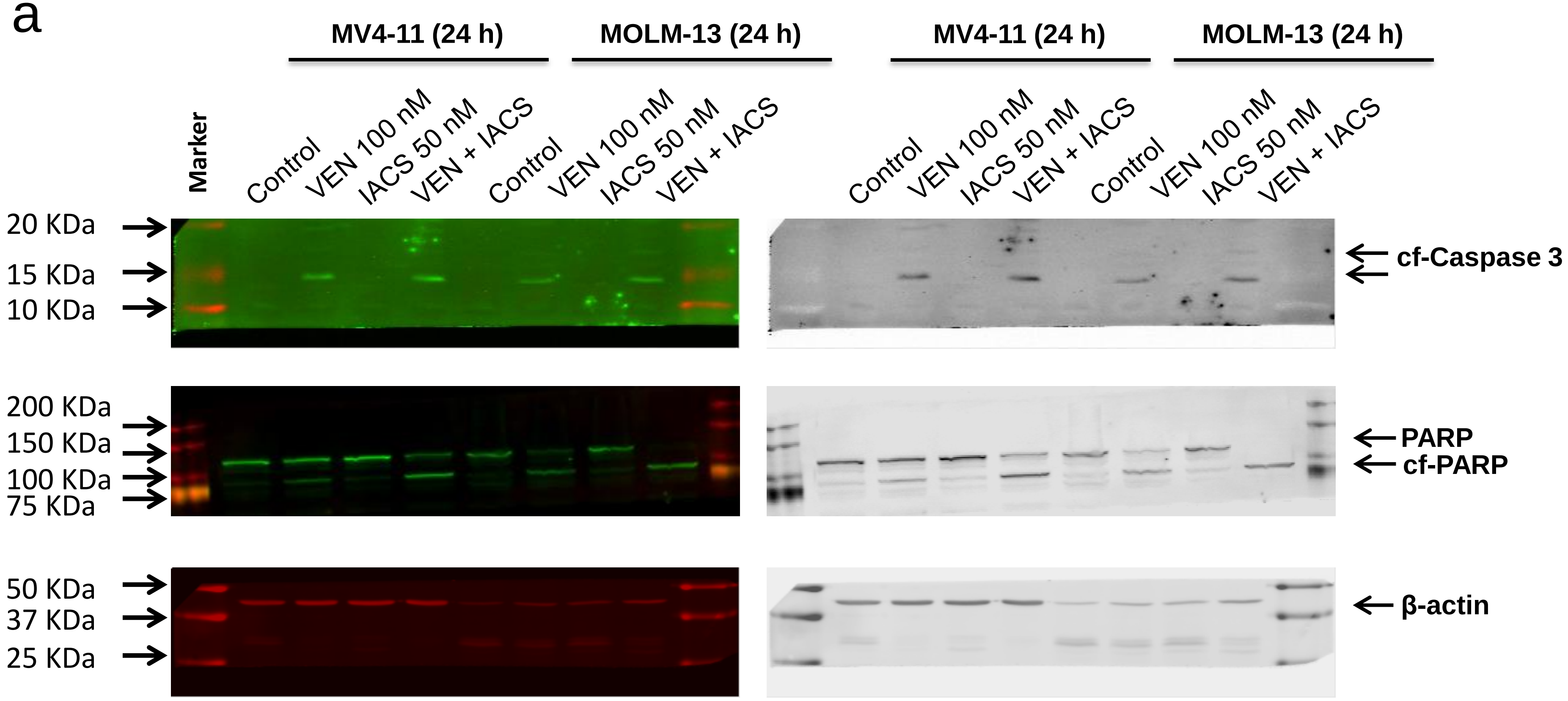

Western blot membranes were cut, based on the prestained ladder, prior to probing. The color image more clearly shows the protein ladder, so it is shown on the left while the black and white image, which is used in the figures, is shown on the right.

Fig. 5A

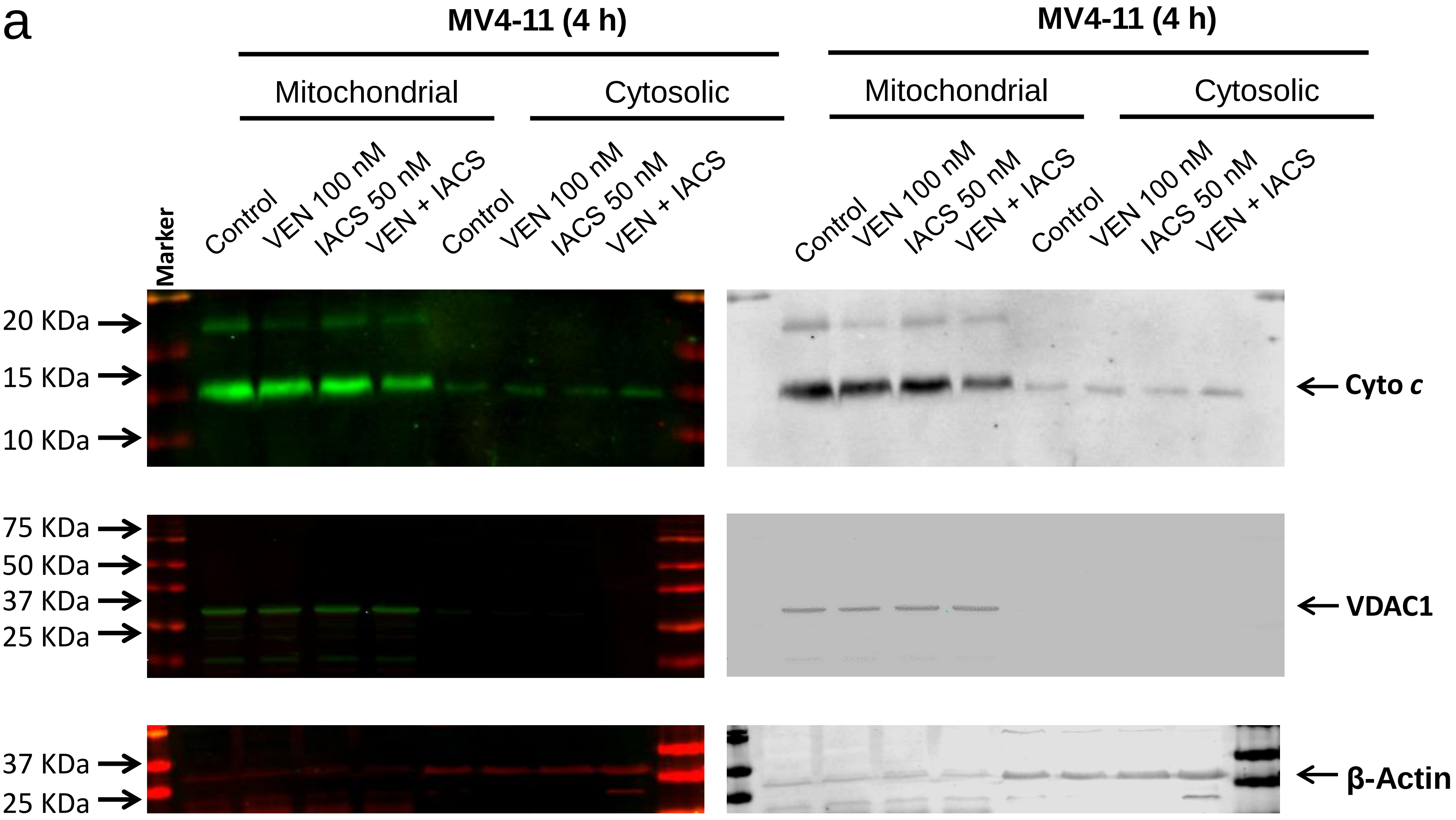

Western blot membranes were cut, based on the prestained ladder, prior to probing. The color image more clearly shows the protein ladder, so it is shown on the left while the black and white image, which is used in the figures, is shown on the right.

Fig. 5A

a

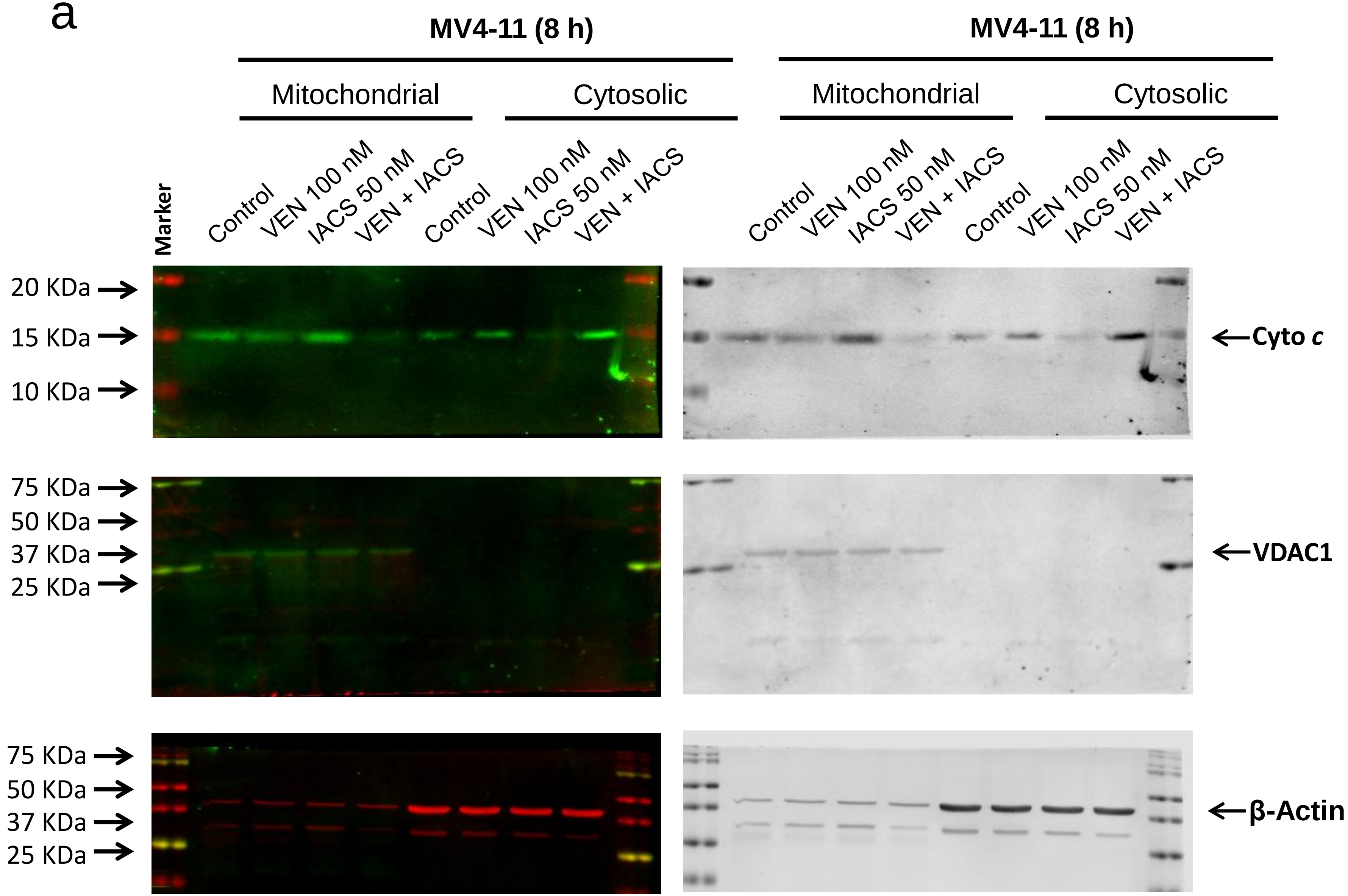

Western blot membranes were cut, based on the prestained ladder, prior to probing. The color image more clearly shows the protein ladder, so it is shown on the left while the black and white image, which is used in the figures, is shown on the right.

Figure 5C

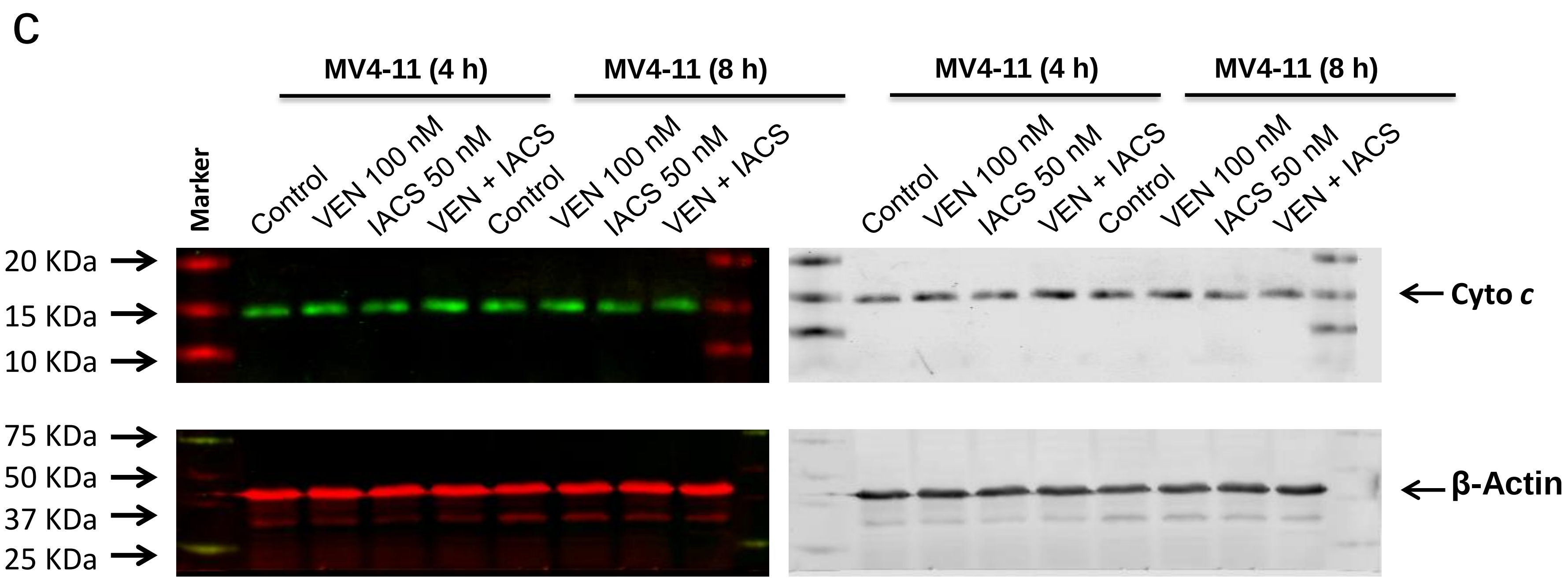

Western blot membranes were cut, based on the prestained ladder, prior to probing. The color image more clearly shows the protein ladder, so it is shown on the left while the black and white image, which is used in the figures, is shown on the right.

Figure 5D

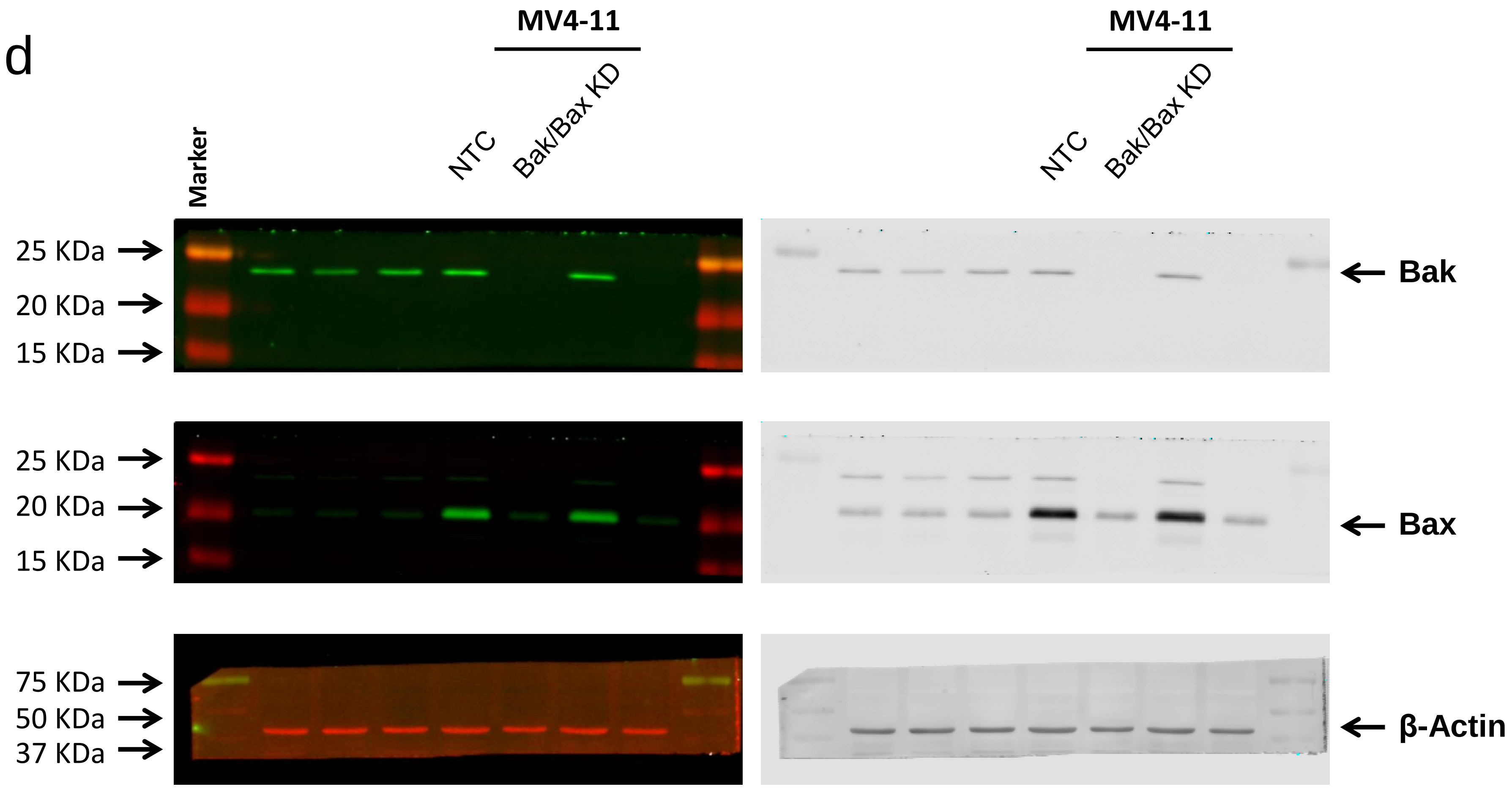

Western blot membranes were cut, based on the prestained ladder, prior to probing. The color image more clearly shows the protein ladder, so it is shown on the left while the black and white image, which is used in the figures, is shown on the right.

Figure 5

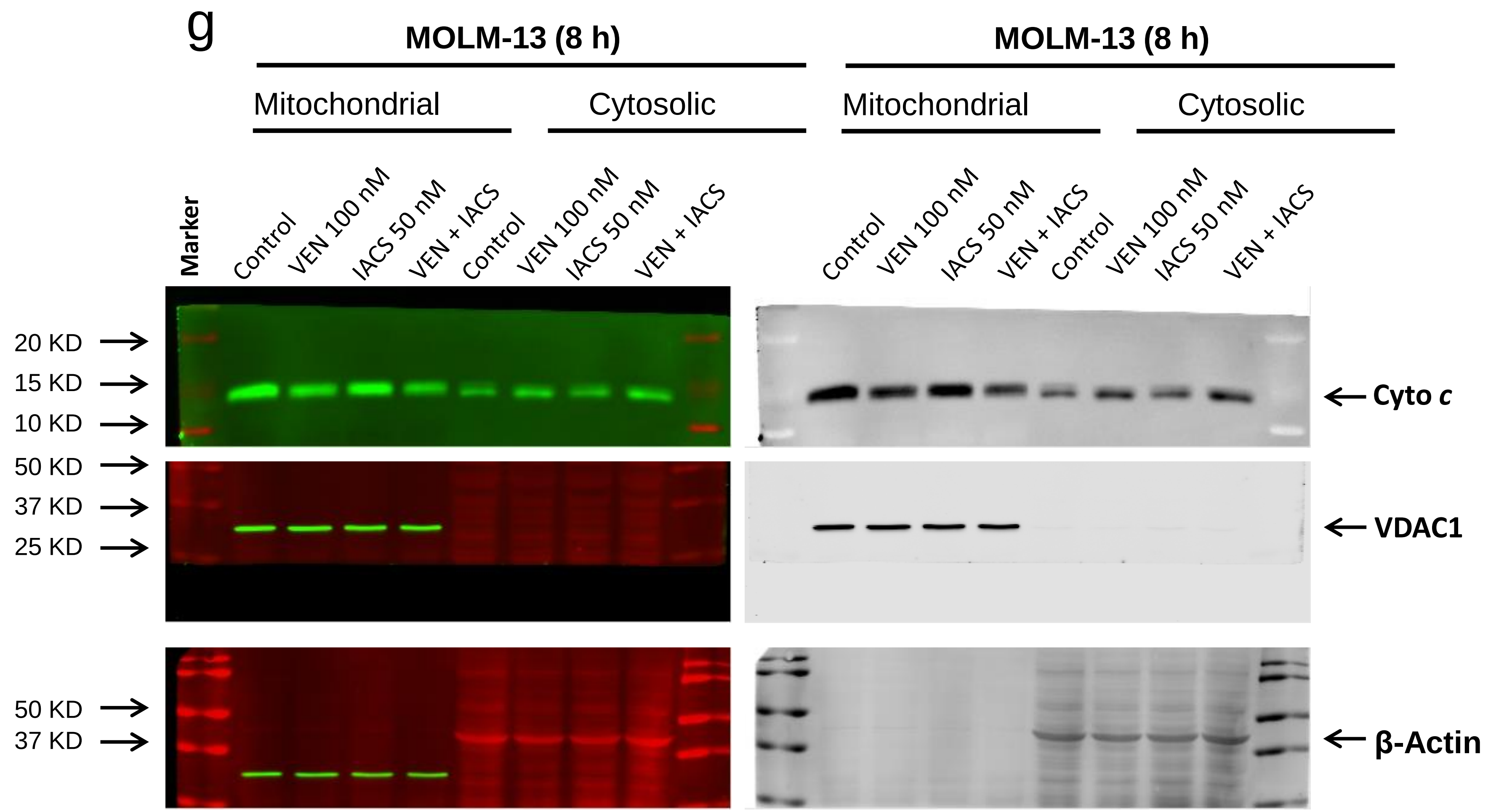

Western blot membranes were cut, based on the prestained ladder, prior to probing. The color image more clearly shows the protein ladder, so it is shown on the left while the black and white image, which is used in the figures, is shown on the right.

Figure 5

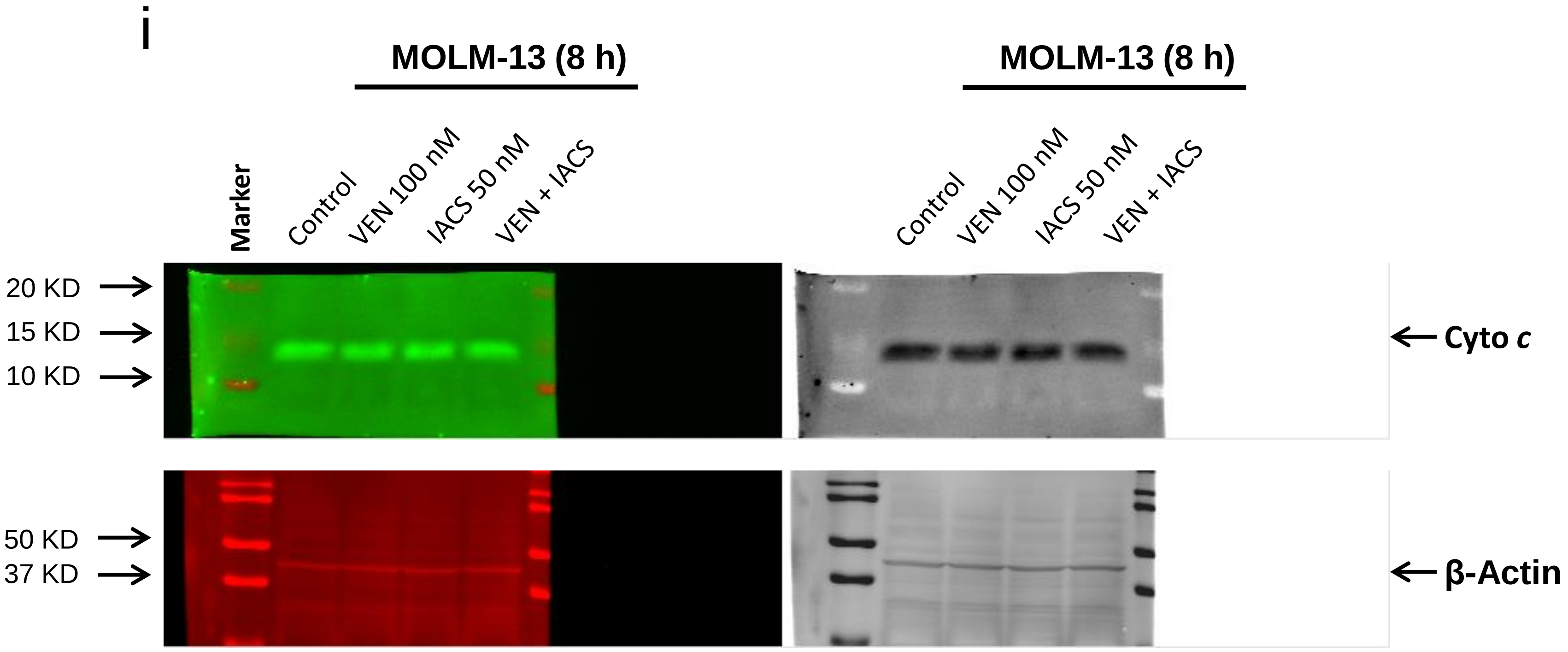

Western blot membranes were cut, based on the prestained ladder, prior to probing. The color image more clearly shows the protein ladder, so it is shown on the left while the black and white image, which is used in the figures, is shown on the right.

Figure 6

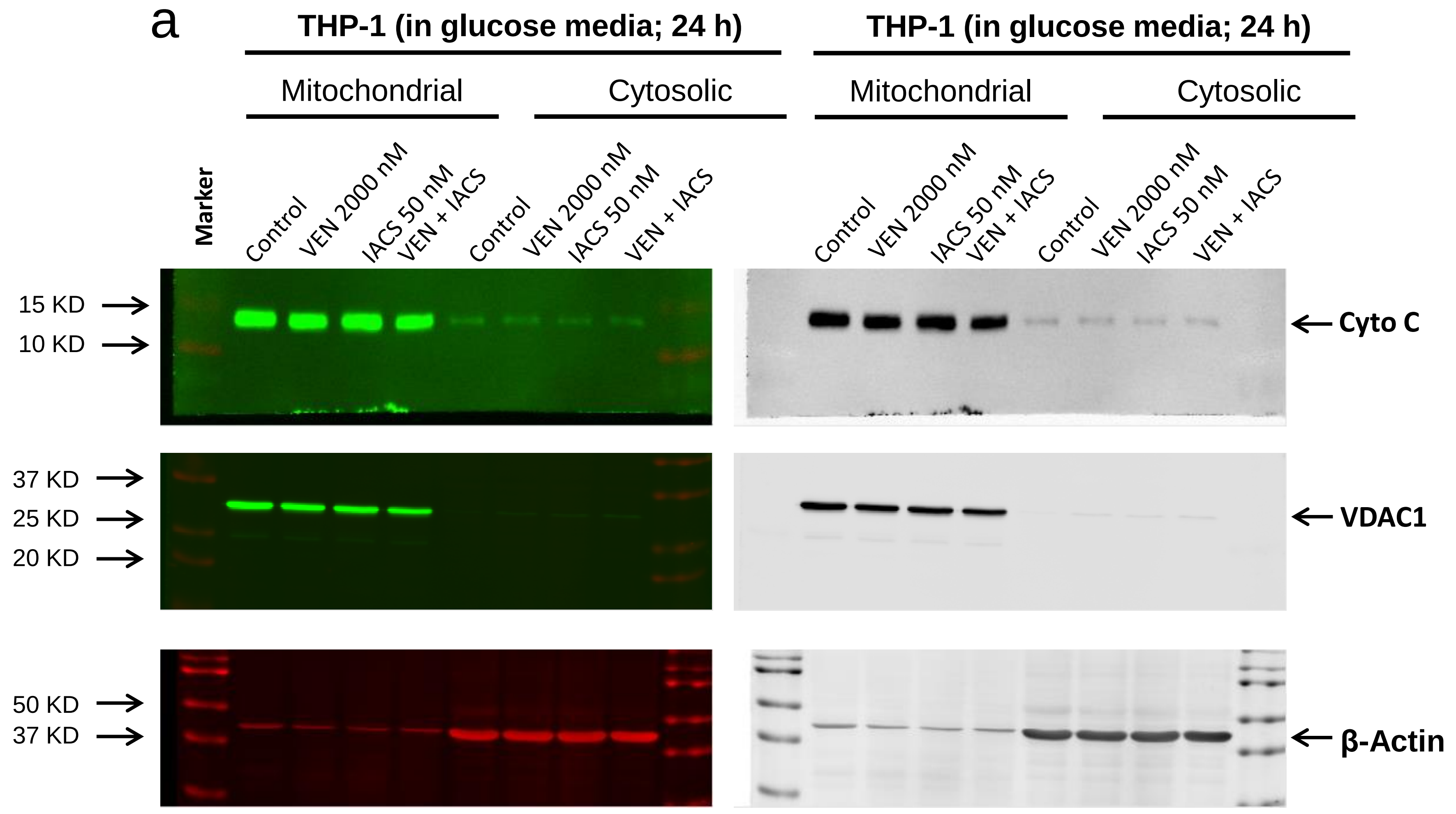

Western blot membranes were cut, based on the prestained ladder, prior to probing. The color image more clearly shows the protein ladder, so it is shown on the left while the black and white image, which is used in the figures, is shown on the right.

Figure 6

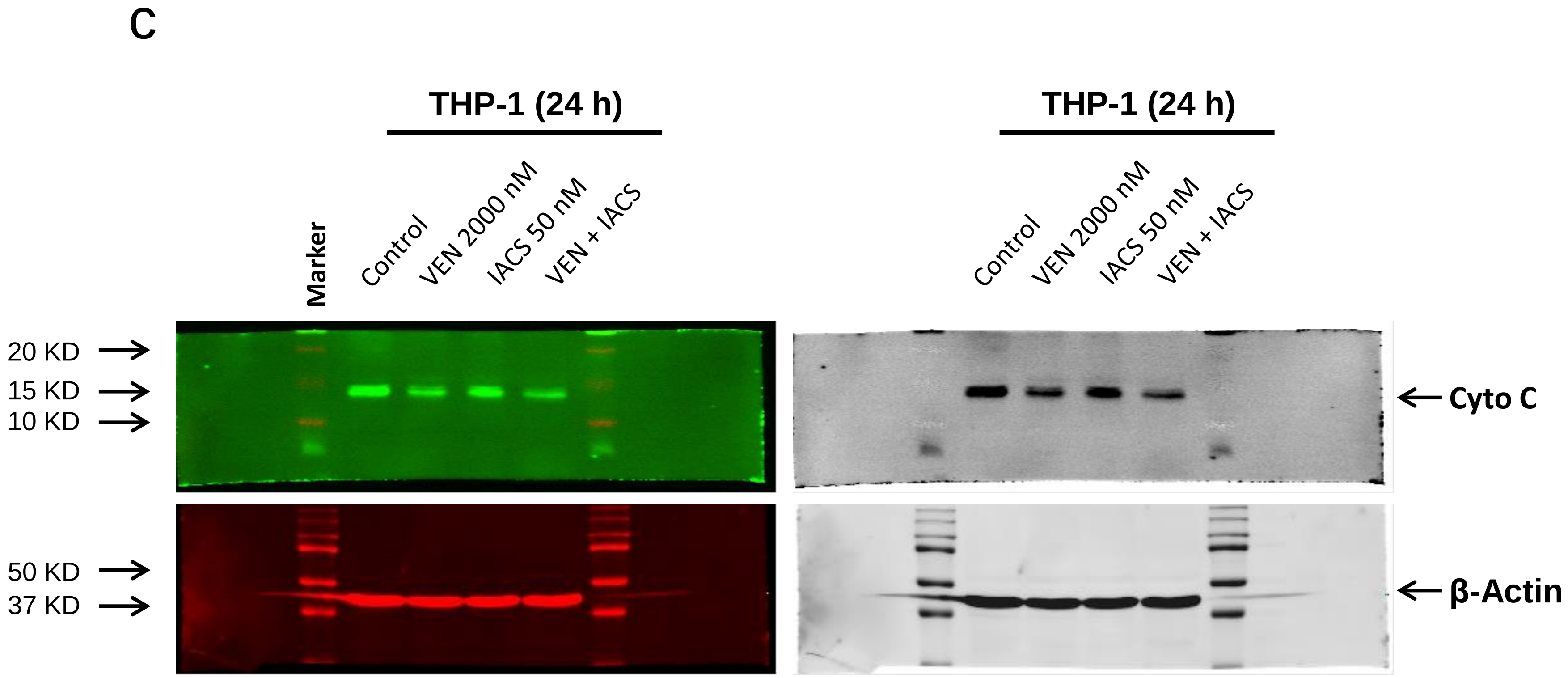

Western blot membranes were cut, based on the prestained ladder, prior to probing. The color image more clearly shows the protein ladder, so it is shown on the left while the black and white image, which is used in the figures, is shown on the right.

Figure 6D

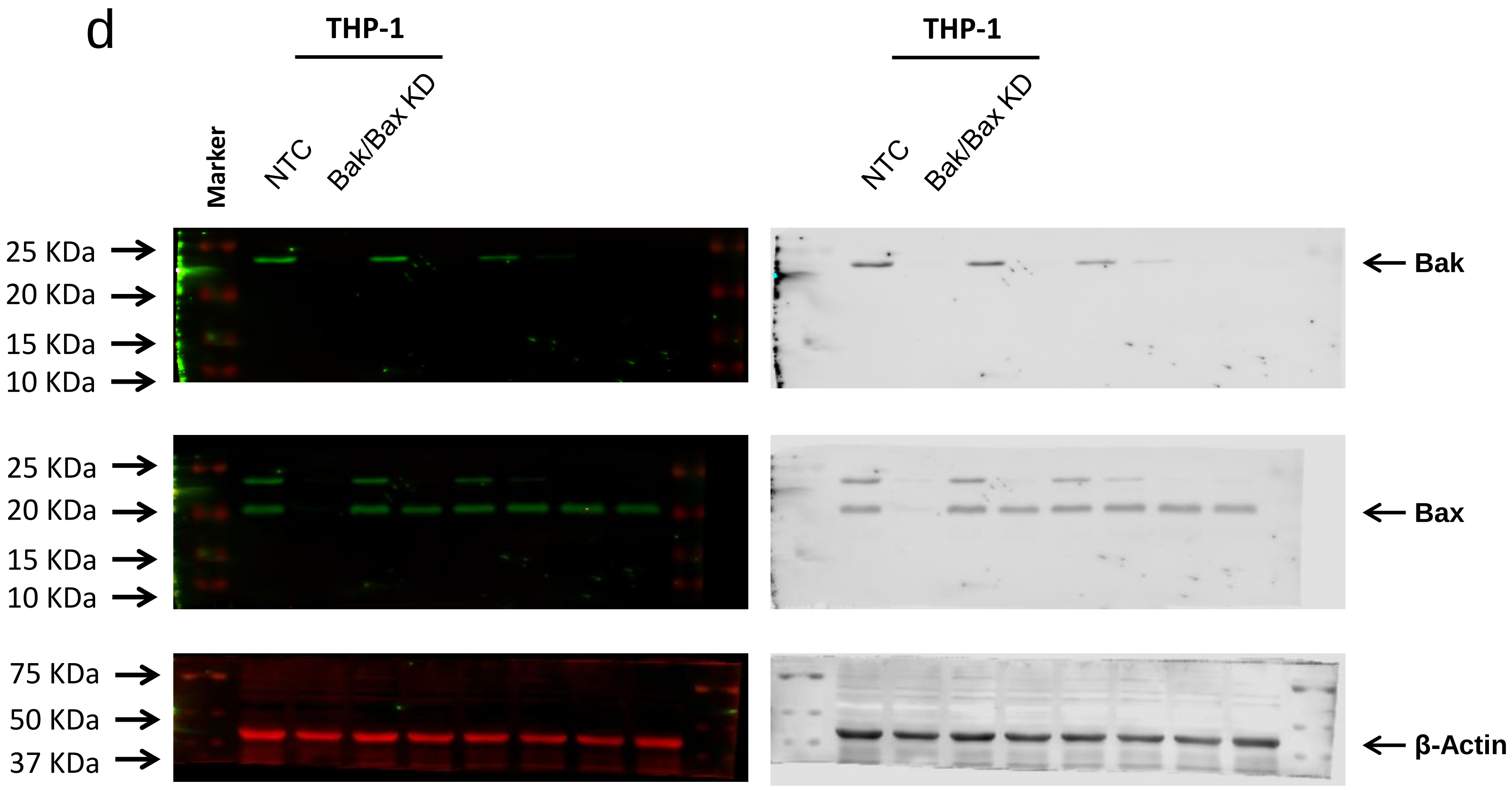

Western blot membranes were cut, based on the prestained ladder, prior to probing. The color image more clearly shows the protein ladder, so it is shown on the left while the black and white image, which is used in the figures, is shown on the right.

Figure 6

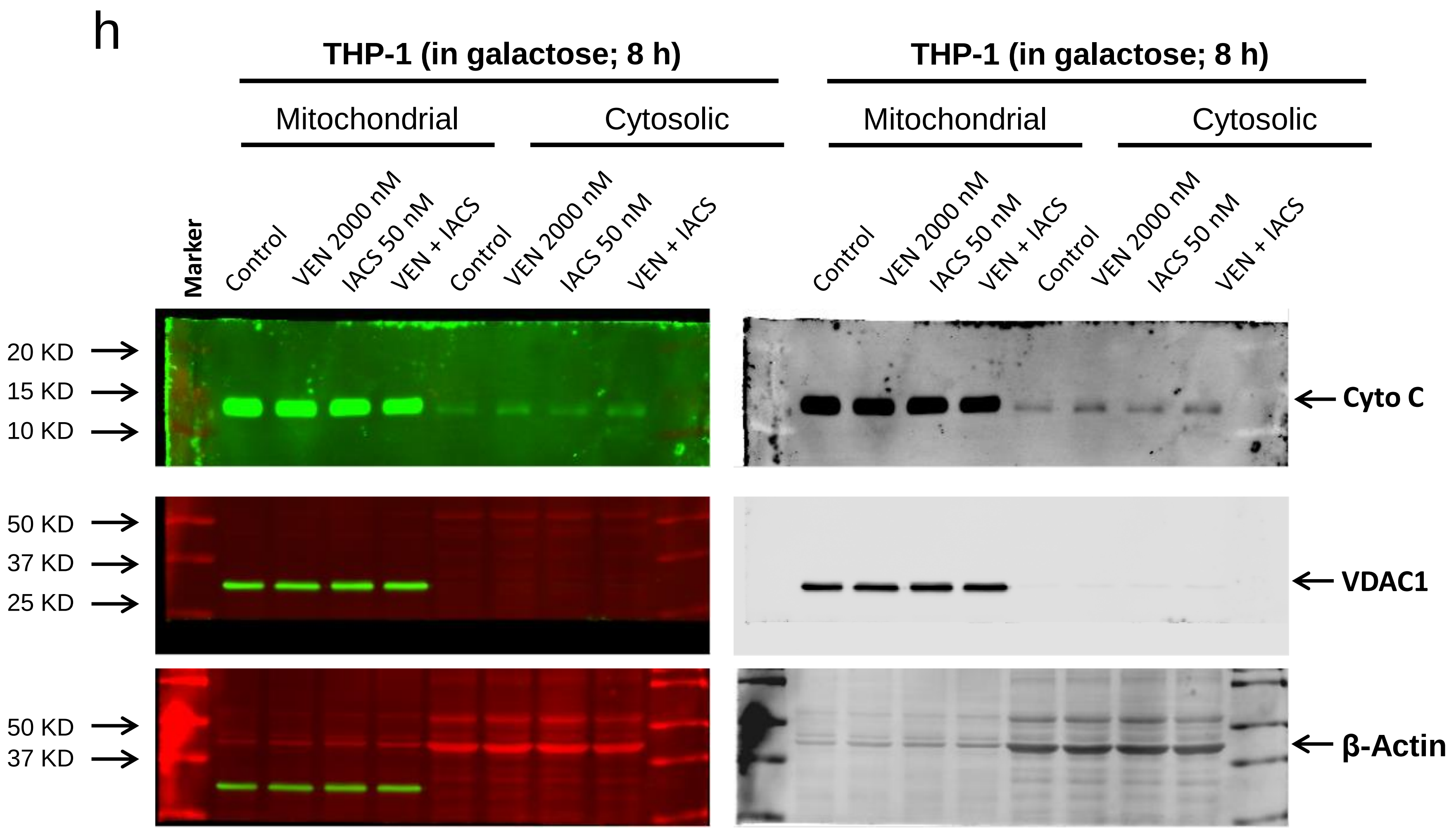

Western blot membranes were cut, based on the prestained ladder, prior to probing. The color image more clearly shows the protein ladder, so it is shown on the left while the black and white image, which is used in the figures, is shown on the right.

Figure 6

j

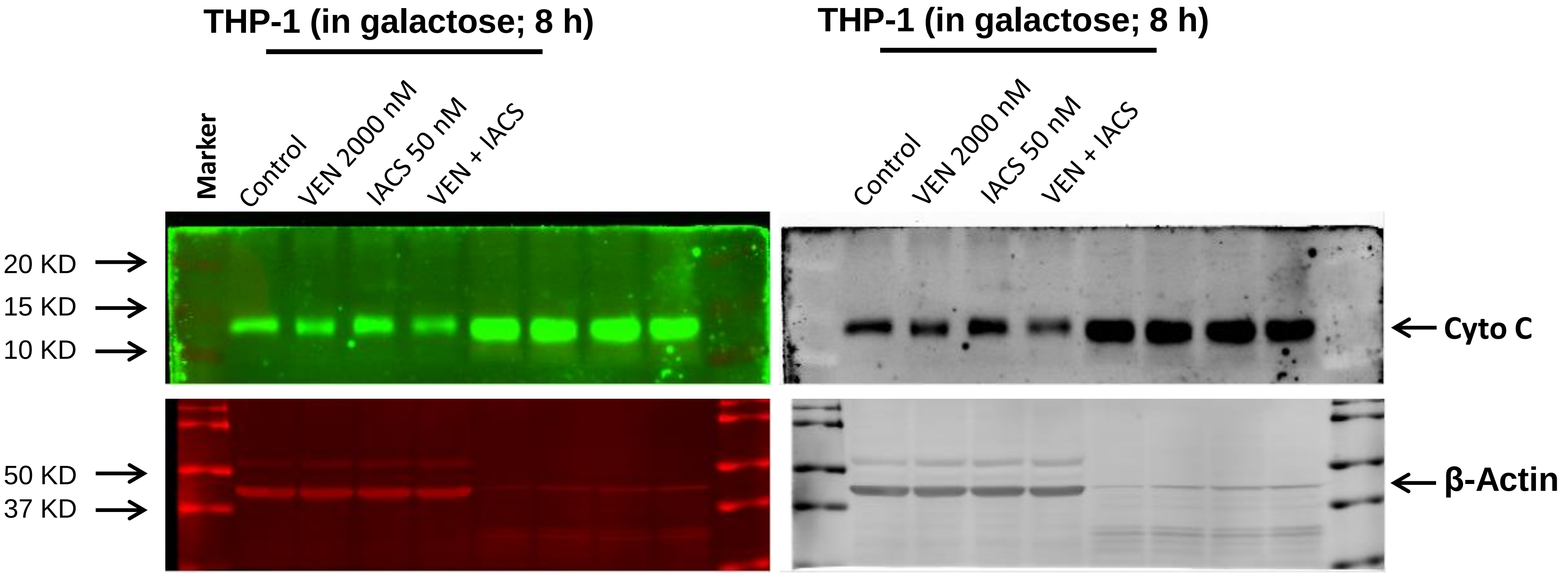

Western blot membranes were cut, based on the prestained ladder, prior to probing. The color image more clearly shows the protein ladder, so it is shown on the left while the black and white image, which is used in the figures, is shown on the right.
